# Supplementary material for: A Likelihood Approach for Real-Time Calibration of Stochastic Compartmental Epidemic Models
Source: PLoS Comput Biol. 2017 Jan 17;13(1):e1005257. doi: 10.1371/journal.pcbi.1005257 (PMC5240920; doi:10.1371/journal.pcbi.1005257)
Supplement: S1 File — (TAR.GZ) [file pcbi.1005257.s014.tar.gz › HSPH_Online-SI-Revision/PF08/n10-mild/n10-mild_PF_table-8.pdf]

| | | | | | | | | | | | | | | | | | | | | | | | | | | | |
|---|---|---|---|---|---|---|---|---|---|---|---|---|---|---|---|---|---|---|---|---|---|---|---|---|---|---|---|
| 1 | R0 | {1.25046, 6.80278} | {1.5667, 19.631} | {{1.49477, 18.7983}, {1.63184, 20.}} | {25.2901, 188.574} | inf | 59. | 494.865 | {415.964, 609.933} | 738.754 | 0.916793 | newly | 76. | 188.26 | {150., 235.} | 147.711 | 0.386222 | Reff | 1.22245 | 1.37789 | {1.33077, 1.44411} | 12.7156 | ar | 3516. | 7113.16 | {6677., 7621.} | 102.308 |
| 2 | R0 | {1.32013, 4.45847} | {1.73047, 18.1308} | {{1.40653, 13.865}, {2.11644, 20.}} | {31.0833, 306.66} | inf | 43. | 329.004 | {199.909, 517.693} | 665.125 | 0.849767 | newly | 76. | 143.08 | {81., 233.} | 89.1316 | 0.241177 | Reff | 1.30046 | 1.60741 | {1.3223, 1.88032} | 24.0522 | ar | 4420. | 7267.84 | {5734., 8599.} | 64.4308 |
| 3 | R0 | {1.31785, 5.36796} | {1.94296, 15.4556} | {{1.27041, 10.7076}, {2.89062, 20.}} | {49.005, 187.922} | inf | 49. | 250.932 | {77.8542, 409.554} | 412.125 | 0.624888 | newly | 66. | 142.4 | {42., 244.} | 129.424 | 0.3242 | Reff | 1.29953 | 1.82687 | {1.22217, 2.51863} | 43.4275 | ar | 4942. | 7212.02 | {4124., 9425.} | 51.7669 |
| 4 | R0 | {1.38801, 6.16031} | {1.69439, 18.5036} | {{1.47547, 13.5649}, {2.06186, 20.}} | {22.1048, 200.368} | inf | 59. | 413.768 | {288.041, 553.574} | 601.303 | 0.835102 | newly | 85. | 175.6 | {115., 224.} | 106.588 | 0.301763 | Reff | 1.35636 | 1.54354 | {1.35949, 1.77483} | 14.3265 | ar | 4790. | 7456.24 | {6325., 8667.} | 55.6626 |
| 5 | R0 | {1.27955, 11.57} | {1.51603, 19.5088} | {{1.50894, 19.4214}, {1.52587, 19.5713}} | {18.4816, 68.6151} | inf | 102. | 536.646 | {529.369, 543.435} | 426.124 | 0.721066 | newly | 64. | 182.33 | {167., 197.} | 184.891 | 0.453775 | Reff | 1.19011 | 0.760362 | {0.755706, 0.764515} | 36.1099 | ar | 3460. | 7110.92 | {7002., 7225.} | 105.518 |
| 6 | R0 | {1.22184, 4.34395} | {2.09704, 14.5347} | {{1.37941, 9.02153}, {3., 20.}} | {71.6565, 234.595} | inf | 20. | 212.986 | {76.2818, 405.223} | 964.929 | 0.937679 | newly | 28. | 150.14 | {38., 309.} | 438.071 | 0.613987 | Reff | 1.21683 | 2.00081 | {1.30638, 2.81256} | 64.5248 | ar | 3524. | 7769.51 | {4991., 9571.} | 120.474 |
| 7 | R0 | {1.23856, 4.59068} | {1.68946, 17.9625} | {{1.38462, 13.1682}, {2.14052, 20.}} | {36.4053, 291.281} | inf | 52. | 336.448 | {188.749, 514.58} | 547.015 | 0.774503 | newly | 78. | 148.04 | {77., 219.} | 92. | 0.248349 | Reff | 1.20995 | 1.56732 | {1.31707, 1.97636} | 29.6193 | ar | 3855. | 7043.4 | {5622., 8761.} | 82.7082 |
| 8 | R0 | {1.34531, 10.6689} | {1.58069, 19.4955} | {{1.5041, 18.6022}, {1.67368, 20.}} | {17.4964, 82.7316} | inf | 98. | 523.334 | {451.232, 603.886} | 434.014 | 0.722513 | newly | 72. | 199.66 | {167., 232.} | 177.306 | 0.437797 | Reff | 1.29352 | 1.38855 | {1.33704, 1.48703} | 7.46626 | ar | 4852. | 7259.01 | {6808., 7681.} | 49.6086 |
| 9 | R0 | {1.31775, 5.19226} | {1.72912, 18.492} | {{1.48593, 13.5549}, {2.07937, 20.}} | {31.2174, 256.146} | inf | 51. | 372.927 | {252.436, 518.187} | 631.229 | 0.845951 | newly | 85. | 158.2 | {102., 243.} | 86.1176 | 0.249714 | Reff | 1.29522 | 1.60062 | {1.38795, 1.88042} | 23.5793 | ar | 4708. | 7578.51 | {6312., 8799.} | 60.9709 |
| 10 | R0 | {1.25916, 6.89207} | {1.589, 18.5441} | {{1.46776, 13.8061}, {1.78952, 20.}} | {26.1953, 169.064} | inf | 121. | 503.146 | {394.919, 642.622} | 315.823 | 0.610394 | newly | 119. | 204.35 | {155., 273.} | 71.7227 | 0.223883 | Reff | 1.17404 | 1.39332 | {1.29103, 1.52432} | 18.6773 | ar | 3823. | 7056.1 | {6408., 7794.} | 84.5697 |
| 11 | R0 | {1.20377, 5.46566} | {1.89058, 17.2583} | {{1.40034, 12.2951}, {2.58295, 20.}} | {57.0553, 215.758} | inf | 61. | 358.391 | {169.147, 663.458} | 487.526 | 0.714384 | newly | 77. | 173.25 | {73., 307.} | 128.117 | 0.312162 | Reff | 1.17392 | 1.75966 | {1.32537, 2.32829} | 49.8965 | ar | 3114. | 7696.66 | {5749., 9505.} | 147.163 |
| 12 | R0 | {1.27878, 2.55804} | {1.92336, 15.5027} | {{1.2414, 10.7187}, {2.71299, 20.}} | {51.8179, 506.04} | inf | 6. | 216.277 | {72.959, 368.434} | 3504.62 | 1.47857 | newly | 12. | 123.17 | {41., 246.} | 926.417 | 0.923613 | Reff | 1.27328 | 1.8163 | {1.20438, 2.47699} | 45.073 | ar | 3930. | 7194.32 | {3906., 9420.} | 85.2667 |
| 13 | R0 | {1.41227, 5.45696} | {1.56675, 19.547} | {{1.49775, 18.8359}, {1.63679, 20.}} | {10.9473, 258.203} | inf | 41. | 492.018 | {420.406, 597.883} | 1100.04 | 1.07541 | newly | 62. | 186.56 | {154., 227.} | 200.903 | 0.473646 | Reff | 1.38233 | 1.37819 | {1.32281, 1.45914} | 2.86368 | ar | 4822. | 7149.76 | {6726., 7641.} | 48.2737 |
| 14 | R0 | {1.32917, 7.27593} | {1.62732, 19.4163} | {{1.52157, 18.2031}, {1.77261, 20.}} | {22.4311, 166.856} | inf | 80. | 501.664 | {411.996, 611.197} | 527.08 | 0.792043 | newly | 96. | 194.21 | {160., 243.} | 102.302 | 0.299524 | Reff | 1.28969 | 1.46156 | {1.36865, 1.58089} | 13.3862 | ar | 4758. | 7543.9 | {6978., 8253.} | 58.5519 |
| 15 | R0 | {1.31629, 9.97481} | {1.70541, 18.7714} | {{1.46437, 14.2704}, {2.04171, 20.}} | {29.5621, 88.1882} | inf | 84. | 410.073 | {295.364, 522.974} | 388.182 | 0.677328 | newly | 70. | 171.3 | {117., 232.} | 144.714 | 0.374409 | Reff | 1.2843 | 1.55258 | {1.34212, 1.78863} | 20.9238 | ar | 4543. | 7494.63 | {6403., 8642.} | 64.9709 |
| 16 | R0 | {1.36735, 5.74166} | {1.63674, 17.7829} | {{1.38429, 13.7378}, {1.91854, 20.}} | {20.2794, 209.717} | inf | 40. | 312.425 | {196.126, 432.691} | 681.063 | 0.86461 | newly | 65. | 139.16 | {82., 196.} | 114.215 | 0.300002 | Reff | 1.34356 | 1.52407 | {1.27931, 1.77616} | 15.2025 | ar | 4516. | 6916.39 | {5550., 8214.} | 53.153 |
| 17 | R0 | {1.27524, 13.2396} | {1.60346, 19.5937} | {{1.52301, 18.8739}, {1.74123, 20.}} | {25.7377, 47.9933} | inf | 39. | 462.438 | {365.574, 561.053} | 1085.74 | 1.06643 | newly | 23. | 177.3 | {137., 220.} | 670.87 | 0.878149 | Reff | 1.26504 | 1.43946 | {1.36836, 1.55765} | 13.788 | ar | 3731. | 7360.59 | {6948., 7981.} | 97.282 |
| 18 | R0 | {1.21884, 7.71545} | {1.61363, 19.2936} | {{1.49184, 18.463}, {1.73949, 20.}} | {32.3907, 150.064} | inf | 68. | 489.893 | {381.04, 589.281} | 620.431 | 0.848186 | newly | 61. | 191.5 | {146., 236.} | 213.934 | 0.485484 | Reff | 1.17923 | 1.45008 | {1.37024, 1.57795} | 22.9683 | ar | 3080. | 7421.53 | {6797., 8180.} | 140.959 |
| 19 | R0 | {1.34879, 6.71464} | {1.79027, 18.1002} | {{1.49787, 13.535}, {2.1488, 20.}} | {32.7317, 169.563} | inf | 96. | 430.716 | {265.619, 626.562} | 348.662 | 0.626759 | newly | 111. | 189.11 | {111., 282.} | 72.4955 | 0.211844 | Reff | 1.29578 | 1.63173 | {1.39089, 1.89683} | 25.9474 | ar | 4585. | 7828.39 | {6576., 8983.} | 70.7391 |
| 20 | R0 | {1.37284, 5.75036} | {1.69619, 17.5725} | {{1.34915, 12.1369}, {2.12858, 20.}} | {24.2708, 205.59} | inf | 46. | 285.509 | {165.914, 444.158} | 520.672 | 0.757428 | newly | 83. | 133.36 | {71., 231.} | 67.2771 | 0.195509 | Reff | 1.34758 | 1.58787 | {1.2902, 1.96086} | 19.9399 | ar | 4750. | 7028.86 | {5328., 8768.} | 48.2232 |
| 21 | R0 | {1.29938, 5.36419} | {1.69956, 17.7422} | {{1.38659, 13.7334}, {2.07536, 20.}} | {30.7977, 230.753} | inf | 36. | 290.813 | {185.283, 437.603} | 707.814 | 0.884013 | newly | 59. | 130.91 | {78., 216.} | 122.22 | 0.319446 | Reff | 1.28145 | 1.58495 | {1.31012, 1.87242} | 23.7723 | ar | 4337. | 7098.82 | {5603., 8510.} | 63.6804 |
| 22 | R0 | {1.31882, 7.47626} | {1.58771, 19.4195} | {{1.50089, 18.5682}, {1.66463, 20.}} | {20.3888, 159.749} | inf | 67. | 471.386 | {384.817, 567.396} | 603.562 | 0.841549 | newly | 68. | 182.76 | {143., 223.} | 168.765 | 0.421999 | Reff | 1.28479 | 1.43191 | {1.34912, 1.51755} | 11.4507 | ar | 3977. | 7317.6 | {6699., 7757.} | 83.998 |
| 23 | R0 | {1.26215, 3.46375} | {1.76391, 16.9282} | {{1.39048, 12.1956}, {2.18962, 20.}} | {39.7712, 388.725} | inf | 5. | 270.982 | {153.531, 403.439} | 5319.63 | 1.7023 | newly | 32. | 132.83 | {69., 216.} | 315.094 | 0.576456 | Reff | 1.25672 | 1.65258 | {1.33911, 1.95943} | 31.607 | ar | 3607. | 7295.09 | {5602., 8842.} | 102.248 |
| 24 | R0 | {1.16712, 4.19464} | {1.63406, 19.2458} | {{1.45343, 17.6066}, {1.84227, 20.}} | {40.0079, 358.82} | inf | 63. | 444.114 | {331.034, 572.169} | 604.942 | 0.838311 | newly | 109. | 174.38 | {120., 236.} | 60.2569 | 0.193299 | Reff | 1.13853 | 1.47974 | {1.33618, 1.7095} | 29.9698 | ar | 3000. | 7282.85 | {6310., 8290.} | 142.762 |
| 25 | R0 | {1.22751, 3.34517} | {2.07403, 14.596} | {{1.36126, 8.3356}, {2.98152, 20.}} | {69.0511, 336.331} | inf | 2. | 187.043 | {68.3761, 346.061} | 9252.13 | 1.88756 | newly | 9. | 132.24 | {33., 254.} | 1369.33 | 1.04316 | Reff | 1.22628 | 1.98542 | {1.29077, 2.81035} | 62.3883 | ar | 3086. | 7686.14 | {5172., 9569.} | 149.128 |
| 26 | R0 | {1.18747, 5.88083} | {1.6167, 19.4973} | {{1.50226, 18.8054}, {1.71548, 20.}} | {36.1469, 231.541} | inf | 134. | 580.068 | {467.517, 703.672} | 332.886 | 0.62814 | newly | 150. | 224.09 | {175., 294.} | 49.4333 | 0.164916 | Reff | 1.11017 | 1.42467 | {1.33987, 1.52841} | 28.3298 | ar | 3614. | 7552.17 | {6945., 8166.} | 108.97 |
| 27 | R0 | {1.29023, 4.76202} | {1.59706, 18.6612} | {{1.43552, 13.5934}, {1.8067, 20.}} | {23.7809, 291.876} | inf | 32. | 414.98 | {312.784, 558.605} | 1196.81 | 1.10166 | newly | 65. | 169.11 | {126., 225.} | 160.169 | 0.404007 | Reff | 1.26391 | 1.45691 | {1.32942, 1.57372} | 15.2705 | ar | 4245. | 7086.88 | {6221., 8071.} | 66.9465 |
| 28 | R0 | {1.2258, 12.9056} | {1.5165, 19.9969} | {{1.50751, 20.}, {1.51705, 20.}} | {23.7148, 54.9476} | inf | 157. | 378.009 | {364.84, 379.728} | 140.77 | 0.380664 | newly | 86. | 124.55 | {109., 140.} | 44.8256 | 0.158857 | Reff | 1.08814 | 0.689016 | {0.68607, 0.691772} | 36.6796 | ar | 3639. | 7737.34 | {7614., 7895.} | 112.623 |
| 29 | R0 | {1.34702, 6.29049} | {1.654, 18.8644} | {{1.46304, 16.2495}, {1.88062, 20.}} | {22.7893, 199.887} | inf | 78. | 387.756 | {273.273, 511.294} | 397.124 | 0.684402 | newly | 109. | 157.98 | {111., 212.} | 46.5138 | 0.155411 | Reff | 1.31765 | 1.52098 | {1.36086, 1.70165} | 15.4931 | ar | 4560. | 7329.9 | {6374., 8326.} | 60.7434 |
| 30 | R0 | {1.37931, 6.78741} | {1.60849, 18.5139} | {{1.43226, 13.7185}, {1.82691, 20.}} | {16.6627, 172.769} | inf | 121. | 422.961 | {304.07, 594.981} | 249.554 | 0.529364 | newly | 153. | 176.31 | {117., 246.} | 26.6078 | 0.101596 | Reff | 1.31324 | 1.45702 | {1.29074, 1.66671} | 11.7948 | ar | 4667. | 7121.83 | {6154., 8270.} | 52.5997 |
| 31 | R0 | {1.16062, 4.47529} | {2.05078, 15.2716} | {{1.3582, 9.96591}, {2.88776, 20.}} | {76.7168, 241.242} | inf | 8. | 209.777 | {63.4389, 424.04} | 2522.21 | 1.32917 | newly | 12. | 134.49 | {38., 297.} | 1020.75 | 0.929823 | Reff | 1.1576 | 1.94904 | {1.25684, 2.66201} | 68.5199 | ar | 3037. | 7671.15 | {5007., 9528.} | 152.748 |
| 32 | R0 | {1.37647, 18.7122} | {1.52965, 19.9354} | {{1.49567, 19.7275}, {1.56153, 20.}} | {11.1286, 6.53688} | inf | 284. | 539.302 | {512.253, 572.777} | 89.895 | 0.278037 | newly | 111. | 178.93 | {162., 194.} | 61.1982 | 0.206179 | Reff | 1.17055 | 0.725851 | {0.71118, 0.742021} | 37.9906 | ar | 4824. | 7557.73 | {7367., 7706.} | 56.6694 |
| 33 | R0 | {1.35084, 6.08841} | {1.66969, 19.0758} | {{1.45143, 16.3738}, {1.93827, 20.}} | {23.6035, 213.313} | inf | 31. | 399.284 | {281.159, 556.536} | 1188.01 | 1.09542 | newly | 44. | 161.69 | {109., 235.} | 267.477 | 0.546351 | Reff | 1.3326 | 1.53684 | {1.38439, 1.76272} | 15.4264 | ar | 4759. | 7361.94 | {6372., 8479.} | 54.6951 |
| 34 | R0 | {1.36912, 6.316} | {1.63266, 18.3322} | {{1.43594, 13.7452}, {1.89503, 20.}} | {19.2489, 190.25} | inf | 47. | 389.641 | {285.154, 526.212} | 729.022 | 0.907089 | newly | 61. | 164.82 | {108., 212.} | 170.197 | 0.415473 | Reff | 1.33681 | 1.49036 | {1.3384, 1.71582} | 12.0636 | ar | 4537. | 7192.05 | {6167., 8355.} | 58.5199 |
| 35 | R0 | {1.35831, 7.09038} | {1.69542, 18.7284} | {{1.45294, 13.9987}, {1.99813, 20.}} | {24.8182, 164.138} | inf | 102. | 427.854 | {293.775, 593.788} | 319.465 | 0.606088 | newly | 115. | 177.31 | {117., 248.} | 54.7217 | 0.172596 | Reff | 1.31498 | 1.55012 | {1.34666, 1.77562} | 18.0263 | ar | 4667. | 7534.13 | {6290., 8607.} | 61.4341 |
| 36 | R0 | {1.28315, 5.89938} | {1.70765, 18.2378} | {{1.38678, 15.2063}, {2.1776, 20.}} | {33.1324, 209.148} | inf | 88. | 299.267 | {178.547, 465.043} | 240.076 | 0.50012 | newly | 139. | 132.29 | {70., 226.} | 34.9137 | 0.156775 | Reff | 1.25672 | 1.59634 | {1.32034, 1.92921} | 27.2591 | ar | 4460. | 7126.23 | {5488., 8738.} | 60.0092 |
| 37 | R0 | {1.19303, 2.75113} | {1.95907, 15.4435} | {{1.25551, 9.00564}, {3., 20.}} | {64.2984, 461.35} | inf | 7. | 235.847 | {90.1015, 385.422} | 3269.24 | 1.45694 | newly | 22. | 144.44 | {42., 272.} | 556.545 | 0.716106 | Reff | 1.1848 | 1.86068 | {1.23054, 2.79234} | 57.3848 | ar | 3142. | 7198.12 | {4408., 9560.} | 129.094 |
| 38 | R0 | {1.36033, 7.8088} | {1.70696, 18.0769} | {{1.43654, 13.9105}, {2.03486, 20.}} | {25.4898, 131.566} | inf | 85. | 364.48 | {254.895, 461.042} | 328.8 | 0.620108 | newly | 88. | 162.07 | {108., 226.} | 84.4659 | 0.25018 | Reff | 1.32551 | 1.58477 | {1.36074, 1.84575} | 19.8497 | ar | 4941. | 7495.39 | {6286., 8678.} | 51.6978 |
| 39 | R0 | {1.27353, 3.70845} | {1.72532, 17.4707} | {{1.41378, 12.8858}, {2.15477, 20.}} | {35.4755, 371.106} | inf | 37. | 303.049 | {179.564, 451.02} | 719.052 | 0.881348 | newly | 69. | 140.43 | {76., 216.} | 103.841 | 0.272571 | Reff | 1.24908 | 1.61772 | {1.30922, 1.97712} | 29.5715 | ar | 3725. | 7234.3 | {5618., 8815.} | 94.2094 |
| 40 | R0 | {1.19983, 12.1486} | {1.48721, 19.6083} | {{1.46996, 19.5163}, {1.49703, 19.6561}} | {23.9517, 61.4041} | inf | 164. | 324.148 | {319.151, 331.2} | 97.651 | 0.295856 | newly | 86. | 108.71 | {98., 119.} | 26.407 | 0.10051 | Reff | 1.07625 | 0.674508 | {0.66262, 0.678854} | 37.3278 | ar | 3619. | 7286.3 | {7171., 7399.} | 101.335 |
| 41 | R0 | {1.20935, 2.67169} | {2.03281, 15.0729} | {{1.28566, 8.98113}, {3., 20.}} | {68.0909, 464.17} | inf | 13. | 183.303 | {77.7052, 316.988} | 1310.02 | 1.07329 | newly | 45. | 125.8 | {37., 245.} | 186.889 | 0.36765 | Reff | 1.20379 | 1.95093 | {1.23411, 2.78203} | 62.2417 | ar | 3429. | 7534.93 | {4534., 9533.} | 119.819 |
| 42 | R0 | {1.23313, 4.74781} | {1.55326, 18.9468} | {{1.41586, 17.3418}, {1.77083, 20.}} | {25.9609, 299.063} | inf | 49. | 448.844 | {360.4, 606.232} | 816.008 | 0.951165 | newly | 79. | 176.83 | {130., 234.} | 123.835 | 0.337545 | Reff | 1.18196 | 1.38208 | {1.281, 1.5295} | 16.9315 | ar | 3835. | 6881.76 | {6164., 7829.} | 79.4462 |
| 43 | R0 | {1.26105, 2.39713} | {1.85592, 16.4422} | {{1.2705, 12.0412}, {2.43257, 20.}} | {47.6537, 585.913} | inf | 6. | 226.719 | {87.7926, 433.709} | 3678.65 | 1.50412 | newly | 32. | 117.18 | {42., 228.} | 268. | 0.494833 | Reff | 1.25298 | 1.74784 | {1.20921, 2.3687} | 41.1313 | ar | 3723. | 7108.44 | {4178., 9000.} | 91.9468 |
| 44 | R0 | {1.36948, 8.88006} | {1.66261, 18.7037} | {{1.44896, 13.7474}, {1.93123, 20.}} | {21.4046, 110.626} | inf | 75. | 403.855 | {299.527, 537.863} | 438.473 | 0.719012 | newly | 68. | 166.32 | {116., 218.} | 144.588 | 0.373965 | Reff | 1.33689 | 1.51798 | {1.3602, 1.72458} | 13.7283 | ar | 4437. | 7336.66 | {6274., 8409.} | 65.3518 |
| 45 | R0 | {1.34882, 3.3791} | {1.99493, 13.4509} | {{1.1546, 6.93514}, {2.86001, 18.8819}} | {52.2671, 298.452} | inf | 2. | 142.698 | {8.16113, 315.841} | 7034.92 | 1.59314 | newly | 4. | 99.18 | {7., 206.} | 2385.5 | 1.1883 | Reff | 1.34828 | 1.89753 | {1.14204, 2.64568} | 46.5151 | ar | 4422. | 6973.24 | {2558., 9257.} | 72.5215 |
| 46 | R0 | {1.23774, 4.34917} | {1.86952, 16.6681} | {{1.25128, 11.4636}, {2.65059, 20.}} | {51.4617, 283.248} | inf | 31. | 250.475 | {87.9426, 507.249} | 707.985 | 0.807139 | newly | 58. | 125.71 | {39., 246.} | 129.397 | 0.301188 | Reff | 1.22252 | 1.75434 | {1.19608, 2.45257} | 44.7343 | ar | 3685. | 7013.74 | {4161., 9354.} | 91.5343 |
| 47 | R0 | {1.34327, 4.78811} | {1.96002, 17.1186} | {{1.43709, 12.4554}, {2.58671, 20.}} | {45.9467, 257.522} | inf | 9. | 280.518 | {144.555, 471.505} | 3016.86 | 1.44999 | newly | 13. | 139.57 | {69., 234.} | 973.615 | 0.981651 | Reff | 1.33642 | 1.85623 | {1.35842, 2.48123} | 39.5311 | ar | 4517. | 7888.38 | {6104., 9450.} | 74.6376 |
| 48 | R0 | {1.24366, 4.25814} | {1.72592, 18.554} | {{1.46738, 14.4132}, {2.08139, 20.}} | {38.7775, 335.73} | inf | 15. | 371.97 | {250.282, 502.78} | 2379.8 | 1.37987 | newly | 19. | 157.82 | {101., 211.} | 730.632 | 0.899152 | Reff | 1.23073 | 1.59974 | {1.38746, 1.8636} | 29.9831 | ar | 3678. | 7595.7 | {6420., 8799.} | 106.517 |
| 49 | R0 | {1.36017, 7.5415} | {1.58849, 19.2382} | {{1.47717, 18.333}, {1.72378, 20.}} | {16.7858, 155.098} | inf | 84. | 554.981 | {457.563, 695.3} | 560.692 | 0.81358 | newly | 104. | 216.64 | {170., 274.} | 108.308 | 0.310969 | Reff | 1.284 | 1.39413 | {1.30127, 1.50243} | 8.74798 | ar | 4178. | 7367.53 | {6793., 8084.} | 76.3411 |
| 50 | R0 | {1.25333, 6.63578} | {1.63175, 19.2182} | {{1.50499, 17.6485}, {1.87424, 20.}} | {30.1932, 189.615} | inf | 101. | 621.713 | {475.906, 789.515} | 515.557 | 0.780634 | newly | 117. | 243.58 | {181., 326.} | 108.188 | 0.307002 | Reff | 1.14855 | 1.41059 | {1.3173, 1.5394} | 22.8147 | ar | 3401. | 7638.85 | {6934., 8482.} | 124.606 |
